# The relationship between calcification inhibitor levels in chronic kidney disease and the development of atherosclerosis

**DOI:** 10.1080/0886022X.2021.1969248

**Published:** 2021-09-28

**Authors:** Can Sevinc, Gulay Yilmaz, Sedat Ustundag

**Affiliations:** a Department of Nephrology, Ataturk University Faculty of Medicine, Erzurum, Turkey; b Department of Transplantation and Nephrology, Acibadem International Hospital, Istanbul, Turkey; c Department of Nephrology, Trakya University Faculty of Medicine, Edirne, Turkey

**Keywords:** Chronic kidney disease, atherosclerosis, vascular calcification inhibitors, carotid intima media thickness

## Abstract

**Aim:**

We aimed to investigate the factors affecting the development of atherosclerosis and the role of calcification inhibitors fetuin-A, matrix-Gla protein (MGP), osteoprotegerin (OPG) in atherosclerosis progress.

**Material and methods:**

The study was planned to investigate the relationship of serum OPG, MGP and fetuin-A levels with the development of atherosclerosis in the stage 2–3–4–5 chronic kidney disease (CKD) patients who did not require dialysis treatment.

**Results:**

32 (17 female, 15 male) healthy individuals and 92 (49 females, 43 males) CKD patients were included. The mean carotid intima-media thickness (CIMT), C-reactive protein (CRP), fetuin-A, OPG and MGP of the two groups were compared statistically. In CKD patients, age, body mass index (BMI), CRP, triglyceride, urea, systolic blood pressure (SBP), fasting blood sugar have a positive linear relationship, fetuin-A, OPG, GFR have a negative linear relationship with CIMT. The mean CIMT, right CIMT, left CIMT, blood urea, CRP, urinary albumin excretion creatinine and age show a negative linear relationship with fetuin-A.

**Conclusion:**

Fetuin-A levels begin to decline from the early stages of CKD and are significantly lower in patients with atherosclerosis as expressed with CIMT. This suggests that fetuin-A may be used as an early marker in CKD for increased cardiovascular risk. Early recognition of these risk factors is important and large-scale studies on vascular calcification inhibitors are needed.

## Introduction

Atherosclerosis associated cardiovascular disease begins from the early stages of chronic kidney disease (CKD) and it is the most important cause of increased morbi-mortality in the CKD process [1]. In addition to the traditional risk factors for atherosclerosis, new risk factors associated with uremia play an important role in the development of early-onset atherosclerosis in the process of CKD, therefore, in recent years, studies are carried out to reveal possible causes. Identifying and controlling these new nontraditional, uremia-related risk factors may contribute significantly to reducing the risk of cardiovascular disease, which is an important cause of morbi-mortality in these patients [2,3]. In studies which were performed with end-stage renal disease (ESRD) patients, it is observed that the calcification occurred in the vascular structures is an important component of the atherosclerosis process. In addition, there was a positive linear relationship between the severity of calcification in atherosclerotic lesions of the coronary or cerebral arteries, the frequency of myocardial infarction and stroke, and the increased morbi-mortality observed in the disease process [4,5]. Vascular calcification was previously considered a passive, degenerative event in the intima and media layer, but it was revealed to be quite complicated in recent years. Bone morphogenetic proteins (BMPs), inorganic pyrophosphate, as well as factors facilitating calcification such as fetuin-A (alpha 2-Heremans–Schmid glycoprotein), matrix-Gla protein (MGP), osteoprotegerin (OPG) levels of calcification inhibitors and also the deterioration of the balance between these factors plays an important role in the increase of vascular calcification observed in uremia and atherosclerosis [6–9]. However, the number of studies investigating the relationship between vascular calcification, atherosclerosis and increased morbi-mortality due to atherosclerosis in ESRD patients is quite small and limited to patients undergoing hemodialysis (HD) [10]. It is known that the development of atherosclerosis and associated morbidity-mortality starts at very early stages in the CKD process. Therefore, patients without a known history of atherosclerotic disease and who developed stage 2–5 CKD due to causes other than Diabetes Mellitus (DM) were included in the study and the development of atherosclerosis was compared with the healthy control group. We aimed to investigate the factors affecting the development of atherosclerosis and the role of calcification inhibitors fetuin-A, MGP and OPG in atherosclerosis progress.

## Material and methods

Our study was planned to investigate the relationship of serum OPG, MGP and fetuin-A levels with the development of atherosclerosis in stage 2–3–4–5 CKD patients who did not require dialysis treatment. The protocol of the study was approved by the Local Ethics Committee (Approval number: TÜBAP-2013/161). Thirty-two (17 female, 15 male) healthy individuals and 92 (49 females, 43 males) CKD patients were included. The healthy control group did not have a history of regular use of medication for any reason and known acute or chronic disease. No pathology was found in the physical examination. Their ages ranged from 19 to 65 years. Glomerular filtration rate (GFR) values were greater than ≥90 mL/min/1.73 m^2^. There was no hematuria and proteinuria. The body mass index (BMI) was <35 kg/m^2^. The group consisted of nonsmoker healthy volunteers. CKD group was non-diabetic, had no acute disease, no history of malignancy and cerebrovascular disease. The average age of the group was 19–65 years, with GFR values ranging from 15–90 mL/min/1.73m^2^, BMI value <35 kg/m^2^; a total of 92 patients were included in the study.

### Biochemical analysis

On the day of the study, the height and weight of healthy controls and individuals in the CKD group were measured and recorded in the morning, following 12 h of fasting. Detailed medical history, chronic diseases, smoking, drugs used in the patient group were also recorded. Complete blood count, fasting blood glucose (FBG), urea, creatinine, uric acid, albumin, total cholesterol, high-density lipoprotein (HDL), low-density lipoprotein (LDL), triglyceride (TG), sodium (Na^+^), potassium (K^+^), calcium (Ca^+2^), phosphate (PO^−4^), iPTH (intact parathormone), Urinary albumin excretion (UAE), urinary protein excretion (UPE) and creatinine excretion were determined from 24-h urine samples. Endogenous creatinine clearance was calculated. GFR of CKD patients was also calculated by using 6 variables extended-MDRD Formula adjusted for body surface area (Ex-MDRD: GFR (mL/min/1.73 m^2^) = 175 × (serum creatinine) − 1.154 × (age) − 0.203 × (BUN) − 0.170 × (albumin) 0.318 × (0.762 women). Fetuin-A, MGP and OPG levels were measured with ELISA kits and Bio-Tek Instruments Microplate EL 309 autoreader device using the micro ELISA method.

### Carotid intima media thickness measurement

Heads of the patients who were placed in the supine position were extended and two measurements were made on the left main carotid artery and the right main carotid artery (1 cm proximal of the bulb). No measurements were made on sites with atheroma plaques. Two echogenic lines between the intima-lumen interface and the media-adventitia interface were evaluated as intima-media thickness of the carotid artery. The mean carotid artery intima-media thickness was calculated by dividing the sum of right and left carotid artery intima-media thickness. Carotid intima-media thickness (CIMT) measurements were measured using the B mode of the Esoat MyLab60 Xvision device.

### Statisical analyiıs

The data of the study were recorded and statistical analysis was performed by using STATISTICA AXA 7.1 (License No: AXA507C775506FAN3). First of all, Kolmogorov–Smirnov test was applied and the appropriateness of the data to normal distribution was investigated. In the study of the differences between the parametric data of the two groups, the Student *t*-Test was used if the data were suitable for normal distribution and the Mann-Whitney U test was used if the data were not suitable for normal distribution. The investigation of the differences between the category data of two separate groups was performed by Chi-square test. In the patient group, the cutoff value of calcification inhibitor levels in determining subclinical atherosclerosis (the power to distinguish those with CIMT <0.75 mm and ≥0.75 mm) was determined by receiver operating characteristics (ROC) analysis. In the study of multiple relationships between CIMT and calcification inhibitors and other parametric data in the patient group; Pearson Correlation Test was used when the data were suitable for normal distribution, Spearman Correlation test was used if at least one of the data was not suitable for normal distribution or if at least one of the data as categorical. Linear regression test (method: Stepwise) was used to investigate the causality relationship between CIMT and calcification inhibitors and other data in the patient group. *p* < 0.05 were considered significant.

## Results

### Comparison of demographic, clinical and laboratory data of healthy control group and CKD group

The demographic, clinical and laboratory data of the healthy control (HC) group and CKD group were compared statistically and the results were shown in Table 1. The mean fetuin-A value was 95.4 ± 16 ng/ml in the HC group and 84.4 ± 22.3 ng/ml in the CKD group. A statistically significant difference was found between the two groups (*p* = 0.02020). The mean OPG value was 12.9 ± 8.6 ng/ml in the HC group and 18.5 ± 10.7 ng/ml in the CKD group, and a statistically significant difference was found between the two groups (*p* = 0.00474). The mean MGP value was 3 ± 2.8 ng/ml in the HC group, 1.5 ± 1.6 ng/ml in the CKD group, and a statistically significant difference was found between the two groups (*p* = 0.00028).

**Table 1.** Comparison of the demographic, clinical and laboratory data of the healthy control group and the CKD group.

Parameters	Healthy control group (*n* = 32)	CKD group (*n* = 92)	*p*
Age (years)	46.2 ± 10.4	46.8 ± 10.3	NS
Gender (female %)	%53	%53	NS
BMI (kg/m^2^)	25.9 ± 4.0	26.3 ± 3.7	NS
SBP (mmHg)	118 ± 9	131 ± 16	<0.0001
DBP (mmHg)	75 ± 6	84 ± 12	<0.0001
FPG (mg/dl)	83 ± 11	88 ± 9	0.0344
Urea (mg/dl)	29.2 ± 8.5	68.9 ± 35.2	<0.0001
Creatinine (mg/dl)	0.7 ± 0.2	2.3 ± 1.4	<0.0001
Uric Acid (mg/dl)	4.8 ± 1.2	6.8 ± 1.4	<0.0001
TG (mg/dl)	152 ± 114	167 ± 110	NS
TC (mg/dl)	176 ± 45	201 ± 49	0.0096
HDL (mg/dl)	48 ± 8	50 ± 11	NS
LDL (mg/dl)	109 ± 38	125 ± 39	0.0435
Albumin (g/dl)	4.4 ± 0.4	4.4 ± 0.4	NS
Na^+^ (mEq/l)	142 ± 2	140 ± 3	0.0003
K^+^ (mEq/l)	4.6 ± 0.4	4.9 ± 0.6	0.0106
Ca^++^ (mg/dl)	9.1 ± 0.4	9.2 ± 0.5	NS
iPO_4_^-^ (mg/dl)	3.3 ± 0.4	3.5 ± 0.7	0.0117
GFR (ml/min/1.73m²)	123 ± 31	44 ± 29	<0.0001
UPE (mg/day)	73 ± 32	994 ± 1234	<0.0001
UAE (mg/day)	11 ± 10	677 ± 905	<0.0001
UNaE (mEq/day)	152 ± 56	142 ± 95	NS
iPTH (ng/ml)	61.2 ± 25.3	154.7 ± 143.2	<0.0001
Hemoglobin (gr/ dl)	13.8 ± 1.4	12.7 ± 1.8	
Platelet (10^3^/μl)	248 ± 53	253 ± 65	NS
Mean CIMT (mm)	0.520 ± 0.052	0.786 ± 0.168	<0.0001
CRP (mg/dl)	0.31 ± 0.22	0.68 ± 0.94	0.0013
Fetuin-A (ng/ml)	95.4 ± 16	84.4 ± 22.3	0.0202
OPG (ng/ml)	12.9 ± 8.6	18.5 ± 10.7	0.0047
MGP (ng/ml)	3.0 ± 2.8	1.5 ± 1.6	0.0003

NS: not significant; BMI: body mass index; SBP: systolic blood pressure; DBP: diastolic blood pressure; FPG: fasting plasma glucose; TG: triglyceride; TC: total cholesterol; HDL: high-density lipoprotein; LDL: low-density lipoprotein; GFR: glomerular filtration rate; UPE: urinary protein excretion; UAE: urinary albumin excretion; UNaE: urinary sodium excretion; iPTH: intact parathyroid hormone; CIMT: carotid intima-media thickness; CRP: C-reactive protein; OPG: osteoprotegerin; MGP: matrix GLA protein.

### Comparison of demographic, clinical and laboratory data of healthy control group and patient groups according to CKD stages

The laboratory data of the HC group, stage 2 CKD group, stage 3 CKD group, stage 4 CKD group and stage 5 CKD groups were statistically compared with the HC group, between themselves and the whole CKD group, the results were given in Table 2. There was no statistically significant difference between the HC group and the groups and between the groups in terms of age and BMI values. When the HC group and CKD groups were compared statistically in terms of mean CIMT values; there was a significant difference between the HC group and the stages 2, 3, 4 and 5 CKD group (*p* < 0.0005). When the CKD groups were compared with each other in terms of mean CIMT values, no statistically significant difference was found. When the HC group and CKD groups were compared statistically in terms of fetuin-a value; there was a significant difference between the HC group and the stage 3 CKD group and the stage 5 CKD group (*p* < 0.05). When the CKD groups were compared between themselves in terms of fetuin-A value, no statistically significant difference was found. When the whole CKD group was compared with the CKD stages in terms of fetuin-a value; there was a significant difference between all CKD groups and the stage 2 CKD group (*p* < 0.05). When the HC group and CKD groups were compared statistically in terms of OPG value; there was a significant difference between the HC group and stage 2, stage 3 and the stage 5 CKD group (*p* < 0.05). When the CKD groups were compared with each other in terms of OPG values, no statistically significant difference was found. When the HC group and CKD groups were compared statistically in terms of MGP value. There was a significant difference between the HC group and stage 2 (*p* < 0.05), stage 3 (*p* < 0.005), stage 4 (*p* < 0.005) and stage 5 (*p* < 0.05). When the CKD groups were compared between themselves in terms of MGP values, no statistically significant difference was found.

**Table 2.** Comparison of demographic, clinical and laboratory data of healthy control group and patient groups according to CKD stages.

	HC (*n* = 32)	Stage 2 (*n* = 26)	Stage 3 (*n* = 34)	Stage 4 (*n* = 23)	Stage 5 (*n* = 8)
Age (years)	46.2 ± 10.4	46.1 ± 11.4	47.4 ± 11.5	46.2 ± 8.1	47.4 ± 7.2
BMI (kg/m^2^)	25.9 ± 4.0	25.6 ± 3	26.1 ± 3.6	26.5 ± 4.2	26.3 ± 3.7
SBP (mmHg)	118 ± 9	127 ± 13**	129 ± 14***	138 ± 18***	133 ± 17***
DBP (mmHg)	75 ± 6	82 ± 12*	83 ± 11**	86 ± 11***	86 ± 13**
FPG (mg/dl)	83 ± 11	87 ± 10	89 ± 8*	88 ± 9	84 ± 14
Urea (mg/dl)	29 ± 9	37 ± 11*	60 ± 23***	99 ± 28^***,a^	119 ± 18^***,a^
Creatinine (mg/dl)	0.7 ± 0.2	1.0 ± 0.2***	1.7 ± 0.6***	3.5 ± 0.9^***,a^	4.7 ± 0.7^***,a,b^
Uric Acid (mg/dl)	4.8 ± 1.2	6 ± 1.4**	6.8 ± 1.3***	7.5 ± 1.3***	7 ± 1.3***
TG (mg/dl)	152 ± 114	157 ± 102	181 ± 130	165 ± 105	152 ± 71
TC (mg/dl)	176 ± 45	214 ± 56**	200 ± 48*	189 ± 41	198 ± 49
HDL (mg/dl)	48 ± 8	53 ± 10	50 ± 11	46 ± 12	51 ± 12
LDL (mg/dl)	109 ± 38	137 ± 43*	128 ± 37*	112 ± 36	115 ± 31
Albumin (g/dl)	4.4 ± 0.4	4.3 ± 0.5	4.5 ± 0.2	4.3 ± 0.3	4.5 ± 0.2
Na^+^ (mEq/l)	142 ± 2	140 ± 2**	140 ± 3**	139 ± 3**	140 ± 3
K^+^ (mEq/l)	4.6 ± 0.4	4.8 ± 0.5	4.9 ± 0.6*	4.8 ± 0.7	5.1 ± 0.9*
Ca^++^ (mg/dl)	9.1 ± 0.4	9.3 ± 0.5	9.4 ± 0.4	8.8 ± 0.5^*,a^	9.2 ± 0.6
iPO_4_^-^ (mg/dl)	3.3 ± 0.4	3.2 ± 0.5	3.4 ± 0.5	3.9 ± 0.6^****,a^	4.3 ± 0.6^***,a^
GFR (ml/min/1.73m²)	123 ± 31	75.5 ± 9.4***	42.8 ± 9.3***	21.7 ± 4.5^****,a^	12.1 ± 2^***,a,b^
UPE (mg/day)	73 ± 32	603 ± 974***	760 ± 1013***	1603 ± 1591^***,a^	1582 ± 1049^***,a^
UAE (mg/day)	11 ± 10	609 ± 1028***	549 ± 721***	879 ± 1098***	966 ± 621***
UNaE (mEq/day)	152 ± 56	146 ± 67	158 ± 135	117 ± 59*	134 ± 38
iPTH (ng/ml)	61 ± 25	83 ± 43*	132 ± 184**	219 ± 86^***,a^	290 ± 145^***,a^
Hemoglobin (g/ dl)	13.8 ± 1.4	13.8 ± 1.1	13 ± 1.7*	11.5 ± 1.7^***,a^	12.1 ± 0.9***
Platelet (10^3^/μl)	248 ± 53	267 ± 68	253 ± 49	258 ± 78	199 ± 59*
Mean CIMT (mm)	0.520 ± 0.052	0.752 ± 0.170***	0.778 ± 0.154***	0.794 ± 0.148***	0.911 ± 0.249***
CRP (mg/dl)	0.31 ± 0.22	0.4 ± 0.3	0.6 ± 0.9*	0.9 ± 1.4**	0.9 ± 0.8***
Fetuin-A (ng/ml)	95.4 ± 16	99.1 ± 7.3	82.1 ± 24.4*	85.3 ± 22.6	77.7 ± 20.2*
OPG (ng/ml)	12.9 ± 8.6	18.3 ± 10.3*	17.9 ± 10.2*	18.2 ± 13.2	22.4 ± 7.3*
MGP (ng/ml)	3.0 ± 2.8	2.0 ± 2.4*	1.4 ± 1.5**	1.3 ± 0.9**	1.1 ± 1.0**

HC: healthy control; NS: not significant; BMI: body mass index; SBP: systolic blood pressure; DBP: diastolic blood pressure; FPG: fasting plasma glucose; TG: triglyceride; TC: total cholesterol; HDL: high-density lipoprotein; LDL: low-density lipoprotein; GFR: glomerular filtration rate; UPE: urinary protein excretion; UAE: urinary albumin excretion; UNaE: urinary sodium excretion; iPTH: intact parathyroid hormone; CIMT: carotid intima media thickness; CRP: C-reactive protein; OPG: osteoprotegerin; MGP: matrix GLA protein.

In comparison of healthy control group and CKD groups *p < 0.05, **p = 0 < 0.005, ***p < 0.0005.

In the comparison of stage 2 and stage 3, stage 4 and stage 5 patients, ^a^*p* < 0.05.

In the comparison of stage 3 and stage 4 and stage 5 patients.

In the comparison of stage 4 and stage 5 patients, ^b^*p* < 0.05.

### Comparison of demographic, clinical and laboratory data of chronic kidney diseases patients with and without subclinical atherosclerosis

The demographic, clinical and laboratory data of the two groups were compared statistically, by dividing the CKD group into two groups as those with CIMT below 0.750 mm (without subclinical atherosclerosis) and those above 0.750 mm (with subclinical atherosclerosis), and the results are shown in Table 3. The mean age of patients without subclinical atherosclerosis and with atherosclerosis was 42 ± 11 years and 51 ± 8 years respectively and a statistically significant difference was found between the two groups (*p* = 0.00002). The mean BMI value (25.1 ± 3.5 kg/m^2^, 27.4 ± 3.6 kg/m^2^ respectively, *p* = 0.00876), the mean SBP value (126 ± 15 mmHg, 136 ± 15 mmHg, respectively, *p* = 0.00409), the mean DBP value (81 ± 11 mmHg, 86 ± 11 mmHg, respectively, *p* = 0.01708). The mean fetuin-a value (91.3 ± 16.7 ng/ml, 78.4 ± 24.9 ng/ml respectively, *p* = 0.02692) of the patients without and with subclinical atherosclerosis were compared.

**Table 3.** Comparison of demographic, clinical and laboratory data of chronic kidney diseases patients with and without subclinical atherosclerosis.

Parameters	CIM*T* < 0.750 mm (*n* = 42)	CIM*T* > 0.750 mm (*n* = 50)	*p*
Age (years)	42 ± 11	51 ± 8	0.0000
BMI (kg/m^2^)	25.1 ± 3.5	27.4 ± 3.6	0.0087
SBP (mmHg)	126 ± 15	136 ± 15	0.0041
DBP (mmHg)	81 ± 11	86 ± 11	0.0171
FPG (mg/dl)	86 ± 9	89 ± 9	NS
Urea (mg/dl)	60 ± 31	76 ± 37	0.0275
Creatinine (mg/dl)	1.9 ± 1.2	2.5 ± 1.4	0.0355
Uric Acid (mg/dl)	6.6 ± 1.2	6.9 ± 1.6	NS
TG (mg/dl)	147 ± 97	184 ± 119	NS
TC (mg/dl)	202 ± 48	200 ± 51	NS
HDL (mg/dl)	53 ± 11	47 ± 11	0.0152
LDL (mg/dl)	125 ± 35	125 ± 42	NS
Albumin (g/dl)	4.4 ± 0.4	4.4 ± 0.3	NS
Na^+^ (mEq/l)	140 ± 3	140 ± 3	NS
K^+^ (mEq/l)	4.9 ± 0.7	4.9 ± 0.6	NS
Ca^++^ (mg/dl)	9.2 ± 0.5	9.2 ± 0.6	NS
iPO_4_^-^ (mg/dl)	3.4 ± 0.6	3.7 ± 0.7	0.0791
GFR (ml/min/1.73m²)	47.9 ± 21.9	40.3 ± 25.1	NS
UPE (mg/day)	1078 ± 1194	924 ± 1273	0.0002
UAE (mg/day)	784 ± 927	584 ± 886	0.0072
UNaE (mEq/day)	131 ± 62	151 ± 116	NS
iPTH (ng/ml)	168 ± 186	144 ± 95	<0.0001
Hemoglobin (g/ dl)	12.8 ± 1.8	12.6 ± 1.8	NS
Platelet (10^3^/μl)	256 ± 65	249 ± 66	NS
Mean CIMT (mm)	0.659 ± 0.083	0.893 ± 0.146	0.893 ± 0.146
CRP (mg/dl)	0.4 ± 0.27	0.91 ± 1.21	0.0072
Fetuin-A (ng/ml)	91.3 ± 16.7	78.4 ± 24.9	0.0269
OPG (ng/ml)	18.7 ± 9	18.3 ± 12	NS
MGP (ng/ml)	1.6 ± 1.8	1.4 ± 1.5	NS

NS: not significant; BMI: body mass index; SBP: systolic blood pressure; DBP: diastolic blood pressure; FPG: fasting plasma glucose; TG: triglyceride; TC: total cholesterol; HDL: high-density lipoprotein; LDL: low-density lipoprotein; GFR: glomerular filtration rate; UPE: urinary protein excretion; UAE: urinary albumin excretion; UNaE: urinary sodium excretion; iPTH: intact parathyroid hormone; CIMT: carotid intima-media thickness; CRP: C-reactive protein; OPG: osteoprotegerin; MGP: matrix GLA protein.

### Evaluation of multiple relationships between carotid intima media thickness and other values in chronic kidney disease patients

**Figure 1.**
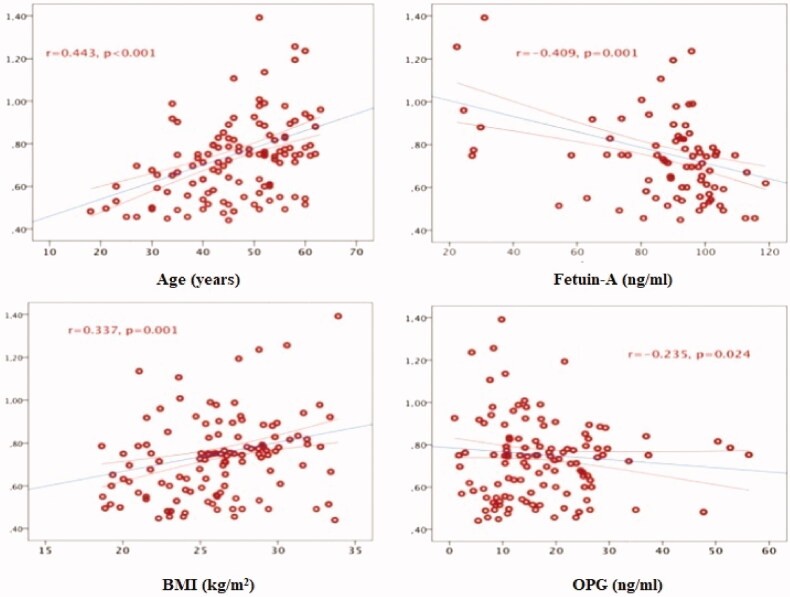
Relationship between CIMT and age, BMI, fetuin-A and OPG.

A positive linear relationship was found between CIMT and age (*r* = 0.493, *p* < 0.001), BMI (*r* = 0.337, *p* = 0.001), CRP (*r* = 0.301, *p* = 0.004), TG (*r* = 0.245, *p* = 0.019), urea (*r* = 0.228, *p* = 0.029), SBP (*r* = 0.212, *p* = 0.043), FPG (*r* = 0.212, *p* = 0.043 in the group of patients with chronic kidney disease. A negative linear relationship was found between CIMT and fetuin-A (*r* = −0.409, *p* = 0.001), OPG (*r* = −0.235, *p* = 0.024) and GFR (*r* = −0.209, *p* = 0.046). Multiple relationships between CIMT and other values are given in Table 4 and Figure 1.

**Table 4.** Evaluation of multiple relationships between carotid artery intima-media thickness and other values in patients with chronic kidney disease.

Parameters	*r*	*p*
Age (years)	0.493	<0.001
BMI (kg/m^2^)	0.337	0.001
CRP (mg/dl)	0.301	0.004
TG (mg/dl)	0.245	0.019
Urea (mg/dl)	0.228	0.029
SPB (mmHg)	0.212	0.043
FPG (mg/dl)	0.212	0.043
Fetuin-A (ng/ml)	–0.409	0.001
OPG (ng/ml)	–0.235	0.024
GFR (ml/min/1.73m²)	–0.209	0.046

### Evaluation of multiple relationships between calcification inhibitor levels and other values in chronic kidney disease patients

In the group of patients with chronic kidney disease; there was a negative linear relationship between fetuin-A level and mean CIMT (*r* = −0.417, *p* = 0.001), right CIMT (*r* = −0.412, *p* = 0.001), left CIMT (*r* = −0.410, *p* = 0.001), urea (*r* = −353, *p* = 0.007), CRP (*r* = −0.322, *p* = 0.014), UPE (*r* = −0.301, *p* = 0.022), creatinine (*r* = −0.277, *p* = 0.035) and age (*r* = −0.262, *p* = 0.047). A positive linear relationship was found between OPG level and left CIMT (*r* = −0.242, *p* = 0.020), mean CIMT (*r* = −0.235, *p* = 0.024) and right CIMT (*r* = −0.222, *p* = 0.034). There was a negative linear relationship between the MGP level and age (*r* = −0.222, *p* = 0.033) and BMI (*r* = −0.215, *p* = 0.040). Multiple relationships between the serum levels of calcification inhibitors and other data are given in Table 5.

**Table 5.** Evaluation of multiple relationships between fetuin-A, osteoprotegerin and matrix GLA protein and other data in patients with chronic kidney disease.

	Parameters	*r*	*p*
Fetuin-A (ng/ml)	CIMT (mm)	−0.417	0.001
	Urea (mg/dl)	−0.353	0.007
	CRP (mg/dl)	−0.322	0.014
	Creatinine (mg/dl)	−0.277	0.035
	Age (years)	−0.262	0.047
Osteoprotegrin (ng/ml)	CIMT (mm)	−0.235	0.024
Matrx GLA protein (ng/ml)	Age (years)	−0.222	0.033
	BMI (kg/m^2^)	−0.215	0.040

### Evaluation of the effect of traditional atherosclerosis risk factors on carotid intima media thickness

In the chronic kidney disease group, the collective contribution of traditional risk factors such as patient age, smoking, body mass index, systolic and diastolic blood pressure, fasting blood sugar, uric acid, total cholesterol, LDL cholesterol, triglyceride, low HDL cholesterol to the increase in CIMT was investigated by applying Multivariate Linear Regression Analysis (model enter). As a result of this analysis, it was seen that the factors listed above (*p* < 0.00001 for the Model) were responsible for the 32.8% carotid artery intima-media thickness increase of the patients’.

### Evaluation of causality relationships between carotid artery intima media thickness and other values

In order to reveal the independent determinant of the increase of CIMT in our CKD group, free from the effects of all other factors, which are shown in Table 3 and related to CIMT were taken and a multivariate linear regression test was applied. In this case (*p* = 0.00015, *R*^2^ = 0.246 for the model), fetuin-A (*p* = 0.007) and patient age (*p* = 0.008) were determined as independent factors leading to the increase in CIMT. Regardless of the effects of all other factors, it was found that each 1 ng/ml decrease in fetuin-A levels increased CIMT by 0.0026 mm, and each 1-year increase in patient age increased CIMT by 0.061 mm (Table 6).

**Table 6.** Evaluation of the independent determinants of the increase in CIMT.

Model	Factor	*p*	Impact (%95 Cl)
*p = 0.00015 *R*^2^=0.246	Fetuin-A	0.007	1 ng/ml Decrease, 0.0026 (0.0007–0.0045) mm CIMT increase
	Age	0.008	1 Year increase, 0.0061 (0.0017–0.0106) mm CIMT increase

CIMT: carotid intima-media thickness.

*Multivariate linear logistic regression analysis (model stepwise).

### The power of fetuin-A level in the CKD group to determine the presence of subclinical atherosclerosis

**Figure 2.**
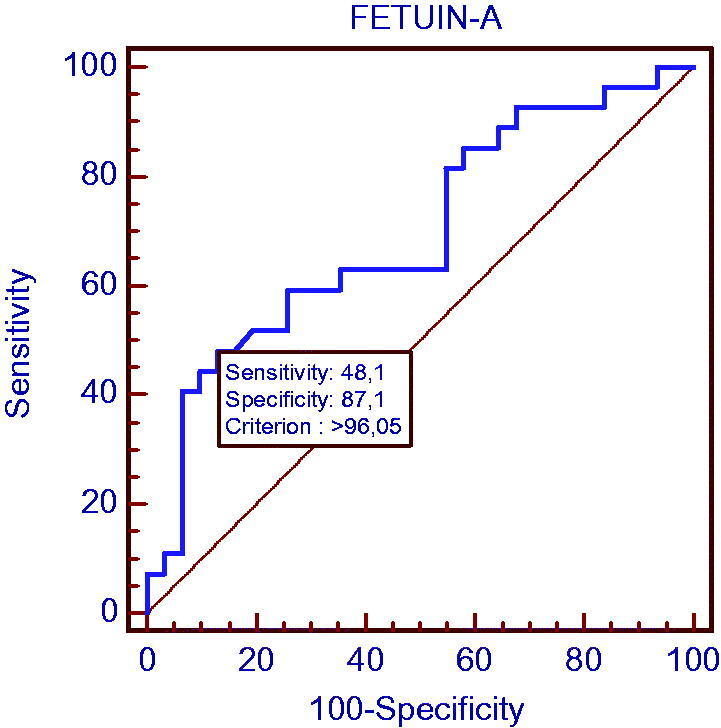
Evaluation of the power of fetuin-A level to determine the presence of subclinical atherosclerosis in the CKD group.

In the group with chronic kidney disease, the power of fetuin-A determining the patients with CIMT >0.750 mm (subclinical atherosclerosis) was investigated by ROC test and the cutoff point was found as 96.05. The sensitivity was 48.1% and specificity was 87.1% at this point. The area under the curve in the ROC analysis was calculated as 0.695 (*p* = 0.006) and is shown in Figure 2.

## Discussion

CKD is a major public health problem worldwide, with a high morbidity-mortality rate and high cost of treatment. In addition to its increasing prevalence, CKD affects public health negatively due to an unacceptably high mortality rate. This elevation in mortality rates is not specific to the advanced stage of kidney disease. Even mild-to-moderate chronic kidney patients are at risk of losing their lives in this process, much more than the chance of progression of kidney disease and the necessity of dialysis treatment [11]. Keith et al., in their study in which they followed 27,998 chronic kidney patients, it was observed that the development of ESRD picture and the need for dialysis in cases with stage 2, 3 and 4 CKD progressed during the 5-year observation period, respectively, by 1.1%, 1.3% and 19.9%; On the other hand, they showed that the loss of life was 19.5%, 24.3% and 45.7% within the same period [12]. It has been determined that the most important cause of increased morbidity-mortality in patients with chronic kidney disease is cardio-cerebrovascular diseases due to athero-arteriolosclerosis [13–15].

CIMT, measured by the ultrasonographic method is considered as a cheap, easy-to-use, repeatable, noninvasive indicator used to evaluate the presence and prevalence of atherosclerosis in epidemiological, clinical and observational studies. A close histological relationship was found between the carotid artery and coronary atherosclerosis in autopsy studies [16,17]. Although coronary angiography provides information about the lesions in the arterial lumen, the development of atherosclerosis with the measurement of CIMT can be considered even in the early stage when anatomic stenosis has not yet occurred and the disorder is limited to the vessel wall only. The very low differences in CIMT evaluation increase its reliability as an indicator of atherosclerosis [18,19]. Studies have shown that the increase in carotid artery CIMT increases the risk of coronary events and related death [20]. CIMT is an independent indicator of cardiovascular mortality in patients undergoing dialysis treatment for end-stage renal disease. Modi *et al.* in their study, 105 patients who underwent coronary angiography and were evaluated with B-mode USG during renal transplant preparation found that coronary artery disease was 73% in patients with a CIMT thickness of 0.750 mm and higher, despite the presence of 7% coronary artery disease in patients with CIMT below 0.750 mm. The results of this study revealed that the CIMT over 0.750 mm was 90% specificity and 73% sensitivity in determining coronary artery disease. It has been suggested that angiography may not be necessary for the investigation of coronary artery disease in patients during the pretransplant period with CIMT <0.750 mm [21]. In our study, CIMT was found to be greater than 0.750 mm as recommended for subclinical atherosclerotic disease threshold in 60% of our patients. Increased CIMT shows a high risk of morbidity-mortality for our non-diabetic, mild-to-middle-stage, early age patients who are not yet known to have a history of the open-cardio-cerebrovascular disease.

The reduction of cardiovascular morbidity mortality in patients with CKD can be achieved by revealing exactly what factors are effective in the development of atherosclerosis and by applying the regulatory interventions for these factors as early as possible. Studies on factors contributing to the increased risk of atherosclerotic disease in patients with chronic kidney disease have largely been carried out with patients undergoing hemodialysis treatment, which constitutes a very small proportion of the chronic kidney patient population, and studies in mild to moderate patients have remained quite limited. In these studies, the cause of increased atherosclerosis development in uremia is not fully elucidated. The presence of traditional atherosclerosis risk factors (age, tobacco use, increased blood pressure, DM, dyslipidemia, obesity) revealed in the Framingham study and other epidemiological studies did not fully explain the increased atherogenicity in CKD and the high morbidity-mortality rate due to atherogenic diseases [22–25]. When we evaluated the combined effects of traditional risk factors such as patient age, smoking, SBP, DBP, FBG, uric acid, LDL, TG levels on CIMT (atherosclerosis development) in our patient group; we found that all these traditional atherosclerosis risk factors contribute approximately 50% of the increase in CIMT in our patients. Our findings are consistent with the findings that increased atherosclerosis and increased morbi-mortality in uremia cannot be explained by traditional risk factors.

In studies performed with ESRD patients, the calcification observed in the vascular structures is an important component of the atherosclerosis process ; There was a positive linear relationship between the severity of calcification in atherosclerotic lesions of the coronary or cerebral arteries, myocardial infarction and stroke, and increased morbi-mortality observed in the disease process. Vascular calcification, which can be defined as bone-like calcium phosphate deposition in the form of bioapatite in the arterial wall; is a clinical condition common in CKD, diabetes mellitus, hypertension and atherosclerosis that causes frequent morbidity and mortality even at a young age. Kramer et al. found that vascular calcification increased in patients with CKD between the ages of 30–65 years compared to the healthy population, and vascular calcification was higher in diabetic CKD patients than in non-diabetic patients [4,5,26–29]. Mechanism of vascular calcification can be explained as; activation of osteogenesis in the vascular wall, deficiency of the factors that prevent calcification, increased bone turnover and disorders in mineral metabolism. Specific calcium regulatory proteins such as fetuin-A, MGP and OPG act as calcification inhibitors with local and systemic effects and play an important role in the development and prevention of uremic vascular calcification. Coronary artery calcification as a result of vascular calcification causes conduction defects, arrhythmia and myocardial fibrosis. In addition, the reduction of fetuin-A, MGP, OPG and OPN in CKD has been shown to contribute to increased vascular calcification [30,31].

Fetuin-A is a glycoprotein with a serum concentration of 0.5–1 g/L, 59 kDa, which is synthesized from the liver in adulthood. It is a member of the cystatin superfamily, a cysteine protease inhibitör. It is a major inhibitor of calcium and phosphate precipitation. Fatal calcifications occur in the kidney, testicles, skin, heart and vessels with congenital calcification in mice undergoing fetuin gene ablation [32]. Fetuin-A stabilizes the crystals of hydroxyapatite to form the particles of calciprotein and cleanse them from the circulation. In HD patients, the serum levels of fetuin-A are low and the activity is decreased. There is a clear correlation between low fetuin-A levels and increased vascular calcification and inflammation in HD patients. An inverse relationship was found between fetuin-A serum level and all-cause mortality, cardiovascular mortality rates and components of MIAC (Malnutrition-inflammation-atherosclerosis-calcification) syndrome in dialysis patients [33–35]. Ketteler et al. found that low-detected fetuin-A levels in patients with chronic renal failure who underwent stable hemodialysis were inversely related to CRP levels, an indicator of inflammation [36]. Kuźniar et al. found that serum fetuin-A levels were lower in all these patient groups compared to healthy individuals in their study evaluating 49 predialysis, 77 hemodialyses, 29 peritoneal dialysis patients and healthy individuals). In addition, they found a negative correlation between the fetuin-A level and coronary calcification and found that fetuin-A plays an important role in the pathogenesis of coronary calcification [37]. In our study, the fact that fetuin-A levels were found to be significantly lower in the patient group with subclinical atherosclerosis compared to the group without subclinical atherosclerosis and significantly lower in stage 3 and stage 5 CKD groups compared to the healthy control group. Fetuin-A was determined as an independent factor leading to the increase in CIMT and it was found that each 1 ng/ml decrease in fetuin-A levels increased CIMT by 0.0026 mm. This predicts that this marker can be used as an early indicator of atherosclerosis.

MGP is the first calcification inhibitor to be detected and expressed from vascular smooth muscle cells and chondrocytes. Studies have shown that MGP inhibits the transformation of mesenchymal cells into osteogenic cells *via* BMP-2 and thus prevents the development of mineralization on the vessel wall. Increased MGP levels have been associated with increased coronary artery calcification and medial vascular calcification resulting in increased cardiovascular mortality [38,39]. Results in the literature regarding the levels of MGP in CKD are variable. When the healthy control group and CKD patients were compared in our study, MGP levels were found to be significantly lower in the stages 2, 3, 4 and 5 compared to the healthy control group. There was no significant difference between the groups with and without subclinical atherosclerosis.

OPG is an inhibitor of osteoblast-derived vascular calcification of the TNF family and completes this by inhibiting osteoclast replacement and activation. High OPG levels are shown to be correlated with the level of calcification in coronary artery disease and coronary artery calcification. In addition, high OPG levels have been associated with an increased risk of cardiovascular death in patients with CKD. An increase of 1 pmol/L in OPG levels has been shown to increase the risk of cardiovascular death by 4%. However, most of the studies with OPG have been conducted in the dialysis population [40,41]. In our study, when the healthy control group and CKD patients were compared, OPG levels were significantly higher in stages 2, 3, 4 and 5 compared to the healthy control group. There was no significant difference between the groups with and without subclinical atherosclerosis in terms of OPG.

In conclusion, our study shows that; In particular, fetuin-A which is a vascular calcification inhibitor, begins to decline from the early stages of CKD and is significantly lower in patients with atherosclerosis. This suggests that fetuin-A may be used as an early marker in CKD with increased cardiovascular mortality. On the other hand, contradictions related to the levels of OPG and MGP in CKD and their role in the development of atherosclerosis continue. The results in our study also support this situation. Reducing mortality and morbidity in CKD primarily depends on reducing the risk of cardiovascular events. Pre-recognition of these risks is important, so large-scale studies on vascular calcification inhibitors are needed.

## Abbreviations

CKD: chronic kidney disease
ESRD: end-stage renal disease
BMPs: bone morphogenetic proteins
MGP: matrix-gla protein
OP G: osteoprotegerin
HD: hemodialysis
DM: diabetes mellitus
GFR: glomerular filtration rate
BMI: body mass index
FBG: fasting blood glucose
HDL: high density lipoprotein
LDL: low density lipoprotein
T G: triglyceride
Na: sodium
K: potassium
P O: phosphate
iP T H: intact parathormone
UAE: urinary albumin excretion
UP E: urinary protein excretion
CIMT: carotid intima-media thickness
ROC: receiver operating characteristics
HC: healthy control
NS: not significant
SBP: systolic blood pressure
DBP: diastolic blood pressure
T C: total cholesterol
CRP: C-reactive protein
